# Texas Medical Association

**Published:** 1873-05

**Authors:** 


*Texas Medical Association.*

This Association met on the 8th of April, at Waco, Texas. It is said to have been the most harmonious and interesting meeting ever witnessed by any members of the Association.

Dr. Wallace read a learned and scientific paper on Hematuria Miasmatica. He also read a good article from Dr. Kilpatrick, of Navasota, on Puerperal Eclampsia.

Dr. Larendon produced a specimen of aneurismal tumor weighing three and a half pounds, and read his article showing that amputation of the thigh was the only means which saved the life of the patient.

Dr. Stuart read two papers; one on Tracheotomy, for removal of foreign substance—operation successful. The second was on Occlusion of Vagina.

Dr. Hudspeth read Dr. Powell's paper, and letters from Dr. Burroughs and himself in reply to Dr. Powell's paper on the Therapeutic Effects of Bromide of Potassa and Fluid Extract of Ergot in the Treatment of Cerebro-Spinal Meningitis, all of which have been referred to the Publishing Committee.

The election of officers resulted as follows: Dr. D. F. Stuart, of Houston, President; Dr. Brown, of Waco, First Vice-President; Dr. Morrison, of Hearne, Second Vice-President; Dr. S. O.

Young, Secretary; Dr. Hamlet, of Waco, Corresponding Secretary; Dr. J. Larendon, Treasurer.

Medicated whisky, as advertised in the newspapers, was attacked by Dr. J. B. Robinson, as referring to the profession for authenticity.

On the 10th, the last day of the session, the Association met at the Waco Female College. The newly-elected President, Dr. D. F. Stuart, called the meeting to order. Music by the choir.

Dr. Flewellen was introduced to the audience, and read an elaborate and learned annual address, in which he took an affectionate farewell from his labors as President of the Association.

Dr. McGregor, of Austin county, was introduced, and read an intelligent and eloquent essay on Medicine and Quackery to the large and appreciative audience of ladies and gentlemen.

Dr. Callaway read a sensible thesis, from the pen of one of the graduating class of his school, on the subject of Hematuria Miasmatica. He also read a paper, showing great research and learning, on Quarantine and Yellow Fever. The Association voted him their thanks for the same.

The Association adjourned, to meet at Dallas the second Tuesday in April, 1864. Peace, harmony and good feeling prevailed in this body during the entire sitting.

We are indebted to Dr. A. R. Kilpatrick for the items above. In this connection, we are specially gratified to be enabled to announce to our readers that Dr. Kilpatrick has consented to become one of our associate editors, to represent, in the pages of the RECORD, his section of the great and growing interest of the medical profession of Texas. Dr. K. is one of the leading minds of the South, an elegant and charming writer, a man of profound thought and large experience, and a ripe scholar. Our readers, we are sure, will rejoice with us in securing the services of his brilliant pen; and our professional brethren in the "Lone Star State"—noted for their highly intelligent and scientific standard—will, we are certain, be more than pleased to know that in Dr. K. they will have another champion and representative worthy of themselves and an honor to their State and profession.

